# Mathematics Is Biology's Next Microscope, Only Better; Biology Is Mathematics' Next Physics, Only Better

**DOI:** 10.1371/journal.pbio.0020439

**Published:** 2004-12-14

**Authors:** Joel E Cohen

## Abstract

Joel Cohen offers a historical and prospective analysis of the relationship between mathematics and biology

Although mathematics has long been intertwined with the biological sciences, an explosive synergy between biology and mathematics seems poised to enrich and extend both fields greatly in the coming decades (Levin 1992; Murray 1993; Jungck 1997; Hastings et al. 2003; Palmer et al. 2003; Hastings and Palmer 2003). Biology will increasingly stimulate the creation of qualitatively new realms of mathematics. Why? In biology, ensemble properties emerge at each level of organization from the interactions of heterogeneous biological units at that level and at lower and higher levels of organization (larger and smaller physical scales, faster and slower temporal scales). New mathematics will be required to cope with these ensemble properties and with the heterogeneity of the biological units that compose ensembles at each level.

The discovery of the microscope in the late 17th century caused a revolution in biology by revealing otherwise invisible and previously unsuspected worlds. Western cosmology from classical times through the end of the Renaissance envisioned a system with three types of spheres: the sphere of man, exemplified by his imperfectly round head; the sphere of the world, exemplified by the imperfectly spherical earth; and the eight perfect spheres of the universe, in which the seven (then known) planets moved and the outer stars were fixed (Nicolson 1960). The discovery of a microbial world too small to be seen by the naked eye challenged the completeness of this cosmology and unequivocally demonstrated the existence of living creatures unknown to the Scriptures of Old World religions.

Mathematics broadly interpreted is a more general microscope. It can reveal otherwise invisible worlds in all kinds of data, not only optical. For example, computed tomography can reveal a cross-section of a human head from the density of X-ray beams without ever opening the head, by using the Radon transform to infer the densities of materials at each location within the head (Hsieh 2003). Charles Darwin was right when he wrote that people with an understanding “of the great leading principles of mathematics… seem to have an extra sense” (F. Darwin 1905). Today's biologists increasingly recognize that appropriate mathematics can help interpret any kind of data. In this sense, mathematics is biology's next microscope, only better.

Conversely, mathematics will benefit increasingly from its involvement with biology, just as mathematics has already benefited and will continue to benefit from its historic involvement with physical problems. In classical times, physics, as first an applied then a basic science, stimulated enormous advances in mathematics. For example, geometry reveals by its very etymology (geometry) its origin in the needs to survey the lands and waters of Earth. Geometry was used to lay out fields in Egypt after the flooding of the Nile, to aid navigation, to aid city planning. The inventions of the calculus by Isaac Newton and Gottfried Leibniz in the later 17th century were stimulated by physical problems such as planetary orbits and optical calculations.

In the coming century, biology will stimulate the creation of entirely new realms of mathematics. In this sense, biology is mathematics' next physics, only better. Biology will stimulate fundamentally new mathematics because living nature is qualitatively more heterogeneous than non-living nature. For example, it is estimated that there are 2,000–5,000 species of rocks and minerals in the earth's crust, generated from the hundred or so naturally occurring elements (Shipman et al. 2003; chapter 21 estimates 2,000 minerals in Earth's crust). By contrast, there are probably between 3 million and 100 million biological species on Earth, generated from a small fraction of the naturally occurring elements. If species of rocks and minerals may validly be compared with species of living organisms, the living world has at least a thousand times the diversity of the non-living. This comparison omits the enormous evolutionary importance of individual variability within species. Coping with the hyper-diversity of life at every scale of spatial and temporal organization will require fundamental conceptual advances in mathematics.

## The Past

The interactions between mathematics and biology at present follow from their interactions over the last half millennium. The discovery of the New World by Europeans approximately 500 years ago—and of its many biological species not described in religious Scriptures—gave impetus to major conceptual progress in biology.

The outstanding milestone in the early history of biological quantitation was the work of William Harvey, *Exercitatio Anatomica De Motu Cordis et Sanguinis In Animalibus (An Anatomical Disquisition on the Motion of the Heart and Blood in Animals)* (Harvey 1847), first published in 1628. Harvey's demonstration that the blood circulates was the pivotal founding event of the modern interaction between mathematics and biology. His elegant reasoning is worth understanding.

From the time of the ancient Greek physician Galen (131–201 C.E.) until William Harvey studied medicine in Padua (1600–1602, while Galileo was active there), it was believed that there were two kinds of blood, arterial blood and venous blood. Both kinds of blood were believed to ebb and flow under the motive power of the liver, just as the tides of the earth ebbed and flowed under the motive power of the moon. Harvey became physician to the king of England. He used his position of privilege to dissect deer from the king's deer park as well as executed criminals. Harvey observed that the veins in the human arm have one-way valves that permit blood to flow from the periphery toward the heart but not in the reverse direction. Hence the theory that the blood ebbs and flows in both veins and arteries could not be correct.

Harvey also observed that the heart was a contractile muscle with one-way valves between the chambers on each side. He measured the volume of the left ventricle of dead human hearts and found that it held about two ounces (about 60 ml), varying from 1.5 to three ounces in different individuals. He estimated that at least one-eighth and perhaps as much as one-quarter of the blood in the left ventricle was expelled with each stroke of the heart. He measured that the heart beat 60–100 times per minute. Therefore, the volume of blood expelled from the left ventricle per hour was about 60 ml × 1/8 × 60 beats/minute × 60 minutes/hour, or 27 liters/hour. However, the average human has only 5.5 liters of blood (a quantity that could be estimated by draining a cadaver). Therefore, the blood must be like a stage army that marches off one side of the stage, returns behind the scenes, and reenters from the other side of the stage, again and again. The large volume of blood pumped per hour could not possibly be accounted for by the then-prevalent theory that the blood originated from the consumption of food. Harvey inferred that there must be some small vessels that conveyed the blood from the outgoing arteries to the returning veins, but he was not able to see those small vessels. His theoretical prediction, based on his meticulous anatomical observations and his mathematical calculations, was spectacularly confirmed more than half a century later when Marcello Malpighi (1628–1694) saw the capillaries under a microscope. Harvey's discovery illustrates the enormous power of simple, off-the-shelf mathematics combined with careful observation and clear reasoning. It set a high standard for all later uses of mathematics in biology.

Mathematics was crucial in the discovery of genes by Mendel (Orel 1984) and in the theory of evolution. Mathematics was and continues to be the principal means of integrating evolution and genetics since the classic work of R. A. Fisher, J. B. S. Haldane, and S. Wright in the first half of the 20th century (Provine 2001).

Over the last 500 years, mathematics has made amazing progress in each of its three major fields: geometry and topology, algebra, and analysis. This progress has enriched all the biological sciences.

In 1637, René Descartes linked the featureless plane of Greek geometry to the symbols and formulas of Arabic algebra by imposing a coordinate system (conventionally, a horizontal x-axis and a vertical y-axis) on the geometric plane and using numbers to measure distances between points. If every biologist who plotted data on x–y coordinates acknowledged the contribution of Descartes to biological understanding, the key role of mathematics in biology would be uncontested.

Another highlight of the last five centuries of geometry was the invention of non-Euclidean geometries (1823–1830). Shocking at first, these geometries unshackled the possibilities of mathematical reasoning from the intuitive perception of space. These non-Euclidean geometries have made significant contributions to biology in facilitating, for example, mapping the brain onto a flat surface (Hurdal et al. 1999; Bowers and Hurdal 2003).

In algebra, efforts to find the roots of equations led to the discovery of the symmetries of roots of equations and thence to the invention of group theory, which finds routine application in the study of crystallographic groups by structural biologists today. Generalizations of single linear equations to families of simultaneous multi-variable linear equations stimulated the development of linear algebra and the European re-invention and naming of matrices in the mid-19th century. The use of a matrix of numbers to solve simultaneous systems of linear equations can be traced back in Chinese mathematics to the period from 300 B.C.E. to 200 C.E. (in a work by Chiu Chang Suan Shu called *Nine Chapters of the Mathematical Art*; Smoller 2001). In the 19th century, matrices were considered the epitome of useless mathematical abstraction. Then, in the 20th century, it was discovered, for example, that the numerical processes required for the cohort-component method of population projection can be conveniently summarized and executed using matrices (Keyfitz 1968). Today the use of matrices is routine in agencies responsible for making official population projections as well as in population-biological research on human and nonhuman populations (Caswell 2001).

Finally, analysis, including the calculus of Newton and Leibniz and probability theory, is the line between ancient thought and modern thought. Without an understanding of the concepts of analysis, especially the concept of a limit, it is not possible to grasp much of modern science, technology, or economic theory. Those who understand the calculus, ordinary and partial differential equations, and probability theory have a way of seeing and understanding the world, including the biological world, that is unavailable to those who do not.

Conceptual and scientific challenges from biology have enriched mathematics by leading to innovative thought about new kinds of mathematics. Table 1 lists examples of new and useful mathematics arising from problems in the life sciences broadly construed, including biology and some social sciences. Many of these developments blend smoothly into their antecedents and later elaborations. For example, game theory has a history before the work of John von Neumann (von Neumann 1959; von Neumann and Morgenstern 1953), and Karl Pearson's development of the correlation coefficient (Pearson and Lee 1903) rested on earlier work by Francis Galton (1889).

**Table 1:**
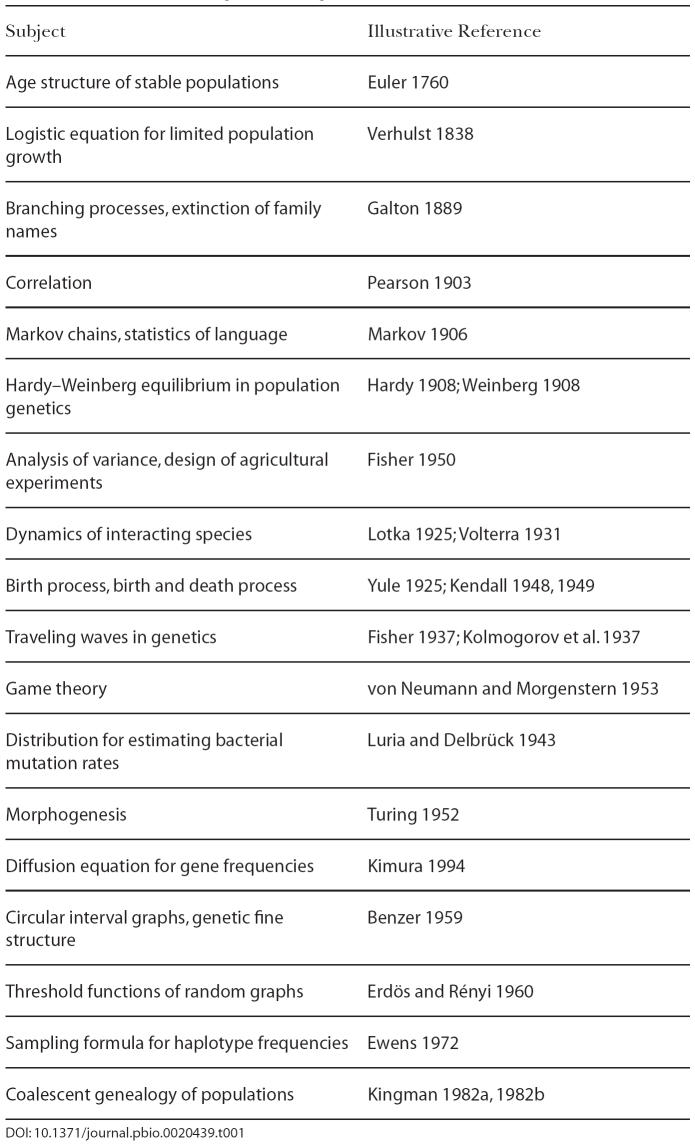
Mathematics Arising from Biological Problems

## The Present

To see how the interactions of biology and mathematics may proceed in the future, it is helpful to map the present landscapes of biology and applied mathematics.

The biological landscape may be mapped as a rectangular table with different rows for different questions and different columns for different biological domains. Biology asks six kinds of questions. How is it built? How does it work? What goes wrong? How is it fixed? How did it begin? What is it for? These are questions, respectively, about structures, mechanisms, pathologies, repairs, origins, and functions or purposes. The former teleological interpretation of purpose has been replaced by an evolutionary perspective. Biological domains, or levels of organization, include molecules, cells, tissues, organs, individuals, populations, communities, ecosystems or landscapes, and the biosphere. Many biological research problems can be classified as the combination of one or more questions directed to one or more domains.

In addition, biological research questions have important dimensions of time and space. Timescales of importance to biology range from the extremely fast processes of photosynthesis to the billions of years of living evolution on Earth. Relevant spatial scales range from the molecular to the cosmic (cosmic rays may have played a role in evolution on Earth). The questions and the domains of biology behave differently on different temporal and spatial scales. The opportunities and the challenges that biology offers mathematics arise because the units at any given level of biological organization are heterogeneous, and the outcomes of their interactions (sometimes called “emergent phenomena” or “ensemble properties”) on any selected temporal and spatial scale may be substantially affected by the heterogeneity and interactions of biological components at lower and higher levels of biological organization and at smaller and larger temporal and spatial scales (Anderson 1972, 1995).

The landscape of applied mathematics is better visualized as a tetrahedron (a pyramid with a triangular base) than as a matrix with temporal and spatial dimensions. (Mathematical imagery, such as a tetrahedron for applied mathematics and a matrix for biology, is useful even in trying to visualize the landscapes of biology and mathematics.) The four main points of the applied mathematical landscape are data structures, algorithms, theories and models (including all pure mathematics), and computers and software. Data structures are ways to organize data, such as the matrix used above to describe the biological landscape. Algorithms are procedures for manipulating symbols. Some algorithms are used to analyze data, others to analyze models. Theories and models, including the theories of pure mathematics, are used to analyze both data and ideas. Mathematics and mathematical theories provide a testing ground for ideas in which the strength of competing theories can be measured. Computers and software are an important, and frequently the most visible, vertex of the applied mathematical landscape. However, cheap, easy computing increases the importance of theoretical understanding of the results of computation. Theoretical understanding is required as a check on the great risk of error in software, and to bridge the enormous gap between computational results and insight or understanding.

The landscape of research in mathematics and biology contains all combinations of one or more biological questions, domains, time scales, and spatial scales with one or more data structures, algorithms, theories or models, and means of computation (typically software and hardware). The following example from cancer biology illustrates such a combination: the question, “how does it work?” is approached in the domain of cells (specifically, human cancer cells) with algorithms for correlation and hierarchical clustering.

### Gene expression and drug activity in human cancer.

Suppose a person has a cancer. Could information about the activities of the genes in the cells of the person's cancer guide the use of cancer-treatment drugs so that more effective drugs are used and less effective drugs are avoided? To suggest answers to this question, Scherf et al. (2000) ingeniously applied off-the-shelf mathematics, specifically, correlation—invented nearly a century earlier by Karl Pearson (Pearson and Lee 1903) in a study of human inheritance—and clustering algorithms, which apparently had multiple sources of invention, including psychometrics (Johnson 1967). They applied these simple tools to extract useful information from, and to combine for the first time, enormous databases on molecular pharmacology and gene expression (http://discover.nci.nih.gov/arraytools/). They used two kinds of information from the drug discovery program of the National Cancer Institute. The first kind of information described gene expression in 1,375 genes of each of 60 human cancer cell lines. A target matrix T had, as the numerical entry in row g and column c, the relative abundance of the mRNA transcript of gene g in cell line c. The drug activity matrix A summarized the pharmacology of 1,400 drugs acting on each of the same 60 human cancer cell lines, including 118 drugs with “known mechanism of action.” The number in row d and column c of the drug activity matrix A was the activity of drug d in suppressing the growth of cell line c, or, equivalently, the sensitivity of cell line c to drug d. The target matrix T for gene expression contained 82,500 numbers, while the drug activity matrix A had 84,000 numbers.

**Figure 1:**
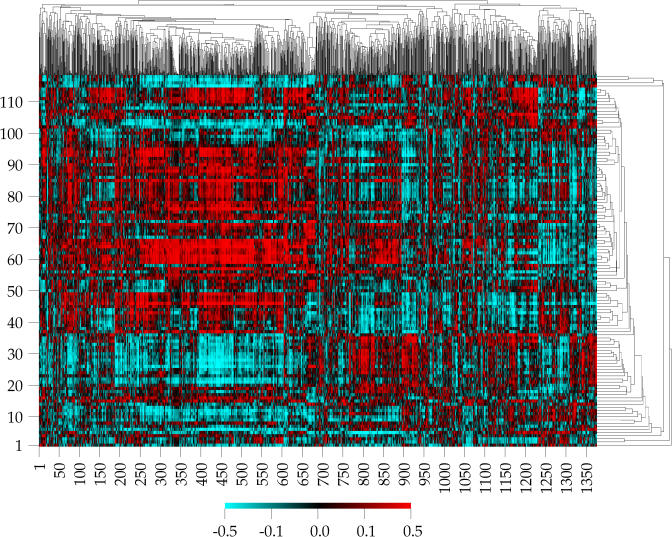
Clustered Image Map of Gene Expression–Drug Activity Correlations Plotted as a function of 1,376 clustered genes (x-axis) and 118 clustered drugs (y-axis). From http://discover.nci.nih.gov/external/CIM_example3/cgi_user_matrix.html. (updated 27 April 2000; accessed 7 October 2004). This image is more recent than the published image (Scherf et al. 2000). Used by permission of John N. Weinstein.

These two matrices have the same set of column headings but have different row labels. Given the two matrices, precisely five sets of possible correlations could be calculated, and Scherf et al. calculated all five. (1) The correlation between two different columns of the activity matrix A led to a clustering of cell lines according to their similarity of response to different drugs. (2) The correlation between two different columns of the target matrix T led to a clustering of the cell lines according to their similarity of gene expression. This clustering differed very substantially from the clustering of cell lines by drug sensitivity. (3) The correlation between different rows of the activity matrix A led to a clustering of drugs according to their activity patterns across all cell lines. (4) The correlation between different rows of the target matrix T led to a clustering of genes according to the pattern of mRNA expressed across the 60 cell lines. (5) Finally, the correlation between a row of the activity matrix A and a row of the target matrix T described the positive or negative covariation of drug activity with gene expression. A positive correlation meant that the higher the level of gene expression across the 60 cancer cell lines, the higher the effectiveness of the drug in suppressing the growth of those cell lines. The result of analyzing several hundred thousand experiments is summarized in a single picture called a clustered image map (Figure 1). This clustered image map plots gene expression–drug activity correlations as a function of clustered genes (horizontal axis) and clustered drugs (showing only the 118 drugs with “known function”) on the vertical axis (Weinstein et al. 1997).

What use is this? If a person's cancer cells have high expression for a particular gene, and the correlation of that gene with drug activity is highly positive, then that gene may serve as a marker for tumor cells likely to be inhibited effectively by that drug. If the correlation with drug activity is negative, then the marker gene may indicate when use of that drug is contraindicated.

While important scientific questions about this approach remain open, its usefulness in generating hypotheses to be tested by further experiments is obvious. It is a very insightful way of organizing and extracting meaning from many individual observations. Without the microscope of mathematical methods and computational power, the insight given by the clustered image map could not be achieved.

## The Future

To realize the possibilities of effective synergy between biology and mathematics will require both avoiding potential problems and seizing potential opportunities.

### Potential problems.

The productive interaction of biology and mathematics will face problems that concern education, intellectual property, and national security.

Educating the next generation of scientists will require early emphasis on quantitative skills in primary and secondary schools and more opportunities for training in both biology and mathematics at undergraduate, graduate, and postdoctoral levels (CUBE 2003).

Intellectual property rights may both stimulate and obstruct the potential synergy of biology and mathematics. Science is a potlatch culture. The bigger one's gift to the common pool of knowledge and techniques, the higher one's status, just as in the potlatch culture of the Native Americans of the northwest coast of North America. In the case of research in mathematics and biology, intellectual property rights to algorithms and databases need to balance the concerns of inventors, developers, and future researchers (Rai and Eisenberg 2003).

A third area of potential problems as well as opportunities is national security. Scientists and national defenders can collaborate by supporting and doing open research on the optimal design of monitoring networks and mitigation strategies for all kinds of biological attacks (Wein et al. 2003). But openness of scientific methods or biological reagents in microbiology may pose security risks in the hands of terrorists. Problems of conserving privacy may arise when disparate databases are connected, such as physician payment databases with disease diagnosis databases, or health databases with law enforcement databases.

### Opportunities.

Mathematical models can circumvent ethical dilemmas. For example, in a study of the household transmission of Chagas disease in northwest Argentina, Cohen and Gürtler (2001) wanted to know—since dogs are a reservoir of infection—what would happen if dogs were removed from bedroom areas, without spraying households with insecticides against the insect that transmits infection. Because neither the householders nor the state public health apparatus can afford to spray the households in some areas, the realistic experiment would be to ask householders to remove the dogs without spraying. But a researcher who goes to a household and observes an insect infestation is morally obliged to spray and eliminate the infestation. In a detailed mathematical model, it was easy to set a variable representing the number of dogs in the bedroom areas to zero. All components of the model were based on measurements made in real villages. The calculation showed that banishing dogs from bedroom areas would substantially reduce the intensity of infection in the absence of spraying, though spraying would contribute to additional reductions in the intensity of infection. The model was used to do an experiment conceptually that could not be done ethically in a real village. The conceptual experiment suggested the value of educating villagers about the important health benefits of removing dogs from the bedroom areas.

The future of a scientific field is probably less predictable than the future in general. Doubtless, though, there will be exciting opportunities for the collaboration of mathematics and biology. Mathematics can help biologists grasp problems that are otherwise too big (the biosphere) or too small (molecular structure); too slow (macroevolution) or too fast (photosynthesis); too remote in time (early extinctions) or too remote in space (life at extremes on the earth and in space); too complex (the human brain) or too dangerous or unethical (epidemiology of infectious agents). Box 1 summarizes five biological and five mathematical challenges where interactions between biology and mathematics may prove particularly fruitful.

**Box 1. Challenges**

**Here are five biological challenges that could stimulate, and benefit from, major innovations in mathematics.**

1. Understand cells, their diversity within and between organisms, and their interactions with the biotic and abiotic environments. The complex networks of gene interactions, proteins, and signaling between the cell and other cells and the abiotic environment is probably incomprehensible without some mathematical structure perhaps yet to be invented.
2. Understand the brain, behavior, and emotion. This, too, is a system problem. A practical test of the depth of our understanding is this simple question: Can we understand why people choose to have children or choose not to have children (assuming they are physiologically able to do so)?
3. Replace the tree of life with a network or tapestry to represent lateral transfers of heritable features such as genes, genomes, and prions (Delwiche and Palmer 1996; Delwiche 1999, 2000a, 2000b; Li and Lindquist 2000; Margulis and Sagan 2002; Liu et al. 2002; http://www.life.umd.edu/labs/Delwiche/pubs/endosymbiosis.gif).
4. Couple atmospheric, terrestrial, and aquatic biospheres with global physicochemical processes.
5. Monitor living systems to detect large deviations such as natural or induced epidemics or physiological or ecological pathologies.

**Here are five mathematical challenges that would contribute to the progress of biology.**

1. Understand computation. Find more effective ways to gain insight and prove theorems from numerical or symbolic computations and agent-based models. We recall Hamming: “The purpose of computing is insight, not numbers” (Hamming 1971, p. 31).
2. Find better ways to model multi-level systems, for example, cells within organs within people in human communities in physical, chemical, and biotic ecologies.
3. Understand probability, risk, and uncertainty. Despite three centuries of great progress, we are still at the very beginning of a true understanding. Can we understand uncertainty and risk better by integrating frequentist, Bayesian, subjective, fuzzy, and other theories of probability, or is an entirely new approach required?
4. Understand data mining, simultaneous inference, and statistical de-identification (Miller 1981). Are practical users of simultaneous statistical inference doomed to numerical simulations in each case, or can general theory be improved? What are the complementary limits of data mining and statistical de-identification in large linked databases with personal information?
5. Set standards for clarity, performance, publication and permanence of software and computational results.
